# Time series momentum: Evidence from the European equity market

**DOI:** 10.1016/j.heliyon.2023.e12989

**Published:** 2023-01-16

**Authors:** Darko B. Vukovic, Salvatore Ingenito, Moinak Maiti

**Affiliations:** a International Laboratory for Finance and Financial Markets, Faculty of Economics, People’s Friendship University of Russia (RUDN University), 6 Miklukho-Maklaya str., 117198, Moscow, Russia; b Geographical Institute “Jovan Cvijic” SASA, Djure Jaksica 9, 11000, Belgrade, Serbia; c Università Cattolica del Sacro Cuore, Faculty for Banking, Finance and Insurance Sciences, Largo A. Gemelli 1, 20123, Milano, Italy; d Independent Researcher, Kolkata, 711112, India

**Keywords:** Time series momentum, Price anomaly, Asset allocation, Autoregression, Factor models

## Abstract

This study empirically analyzes time series momentum (TSM) in the European equity market between 2000 & 2020. The study produces additional evidence on TSM where a significant and persistent market price anomaly enables investors to earn abnormal returns. To achieve this goal the present study implements a pooled autoregressive model to test the predictability power of European equity indices of future returns. The results indicate that strategies based on TSM are in line with the discussed literature and enable market agents to earn returns above the market (0.71% per month) by using a six-factor model.

## 1 Introduction

Moskowitz et al. [1] introduced a relatively new form of momentum effect termed as “Time Series Momentum” (TSM) of market inefficiency. Thereafter, a huge body of literature investigate this phenomenon of market inefficiency across different periods and/or diverse asset classes [2–3]. Whereas cross-sectional momentum, the empirical evidence of time series momentum suggests looking back 1 to ‘*n’* months securities' own past returns, buy winner assets and short loser assets, keep the position for the following 1 to ‘*n’* months and then, close the position [1]. The design of this tactical asset allocation strategy clearly ignores cross-sectional means of peer assets which is pivotal in the traditional momentum strategy, i.e., cross-sectional momentum. The evidence provided by Moskowitz et al. [1], He and Li [2], D’Souza et al. [4], Hurst et al. [5], Georgopoulou and Wang [6], Liu et al. [3], & others show that trading strategies based on TSM enable to enhance portfolios' alpha challenging therefore the ‘Efficient Market Hypothesis’. Returns from these strategies do not load significantly on other risk factors such as the Fama & French five factors [1]. Market efficiency *per se* is not testable and TSM as a price anomalxy is still doubtful. However, an interest survey conducted by Schwert [7] finds that when a market anomaly is documented, it tends to vanish either because of a sample bias or because market agents trade accordingly resulting in the disappearance of the anomaly. In contrast, time series momentum seems not to have vanished since its inception. Perhaps, we are truly in front of a market price anomaly which systematically enables us to earn extra profit above the overall market. The studies done on time series momentum suggest that this phenomenon is persistent across different asset classes, markets, countries, and periods. However, the literature on TSM seems to lack recent and specific empirical evidence related to the European equity market.

Is TSM a significant and persistent market price anomaly in Europe throughout the first 20 years of 2000s? If so, does it enable investors to earn abnormal returns? The topic has a relevance in both theoretical and practical aspects. Adding one more piece of evidence on efficient market hypothesis and price anomalies theory would provide global investors and policy makers with some additional information to beat the market. To examine it this study demonstrates the existence of time series momentum in the European equity market over longer durations. The study tests propositions from Moskowitz et al. [1], He and Li [2], D’Souza et al. [4], Hurst et al. [5], Georgopoulou and Wang [6] and Liu et al. [3], by implementing a pooled autoregressive model using the twenty-four main equity indices across Europe. We find strong evidence in favor of TSM since the 1-to-12 monthly return lags exhibits highly significant and positive coefficients, proving that assets' own past returns are positive predictors of future returns. To provide the significance of time series momentum, the tactical asset allocation idea is to go long (short) when the return over the lookback period (k) is positive (negative), hold the position for one month (h) to close it immediately after. We consequently structure and backtest a trading strategy in line with TSM logic to assess its performance and quantify the extra profit deriving from this ‘price anomaly’. It turns out that all trading strategies, i.e., using different lookback periods (i.e., 1-to-12-month lags), generate by far better performances in mean-variance terms relative to a simple long passive strategy (e.g., B&H). Also, if we implement TSM strategy over a non-diversified portfolio, Sharpe ratio appears positive and large. For each European country, Sharpe ratio from TSM is dramatically higher than how it is from B&H strategy. Furthermore, the study self-selects one of the 12 strategies (k = 12 and h = 1) to gauge TSM’s abnormal performance and control for passive exposure. First, alpha is found to be significant at 0.71% per month while it is not for a long passive strategy. Second, TSM strategies do not exhibit exposure to the Stoxx600 as overall European market proxy and to the six-factor model (Fama & French five factors plus momentum factor), while the B&H strategy loads significantly on most of these factors. TSM excess returns seem to be significantly related to the cross-sectional European momentum factor. In the light of these findings, we draw a scatter plot using the excess returns of the Stoxx600 and of the TSM strategy. The trend looks like a straddle payoff, which means that during more pronounced market movements, TSM strategies earn extra-profit. In general, the study create portfolio of 12-month lag returns of the 24 most traded European equity indices to predict their future returns, with the goal to quantify the performance of a time series momentum strategy and with look back periods equal to the number of month lag returns that exhibit significant coefficient (i.e., the first 12-month lags, and one month of holding period). Recently TSM not only gained attention across the global financial market investors but also gained equal attention among the crypto investors [3,[8], [9], [10], [11], [12], [13]]. This study aims to test the evidence on TSM in the European equity market.

The present study has six sections which are presented as follows: Following the introductory section, section 2 discusses the literature through three parts. The first part discusses efficient market hypothesis and price anomalies with the greatest reference to Fama [14], Jensen [15] and Schwert [7]. The second part discusses the most relevant literature from cross sectional momentum to time series momentum. Within this part, there is also a discussion of the studies of De Bondt and Thaler [16], Jegadeesh and Titman [17], Conrad and Kaul [18] and Moskowitz et al. [1] and their theoretical models. The third part discusses and criticizes the time series momentum models from Moskowitz et al. [1], to authors who have given a critical review in recent years, including: He and Li [2], D’Souza et al. [4], Georgopoulou and Wang [6], Huang et al. [19], Pitkäjärvi et al. [20] and Liu et al. [3] and others. Section 3 covers the sample and methodology discussed in section 2. The dataset is obtained from Bloomberg terminal including national equity indices from the European market. Motivated by Moskowitz et al. [1], the study uses a pooled autoregression model suggested by Davidson and MacKinnon [21] for the dynamics of change to show empirical evidence on the predictability power of equity indices' own past returns. Similar model is also used by D’Souza et al. [4] and Pitkäjärvi et al. [20] in their studies. Next, we use a portfolio construction and the tactical asset allocation suggested by Moskowitz et al. [1], and Pitkäjärvi et al. [20] to present several strategies based on different lookback periods (k) and one month holding period (h). Lastly, the study in the section uses multi factor models to test abnormal performance from the time series momentum strategy. The results of the study are presented in section 4, including: evidence of 1-Year time series momentum (based on Moskowitz et al. [1] and Pitkäjärvi et al. [20], backtest of TSM strategies (suggested previously by Brooks and Kat [22], performance of the conventional TSM strategy suggested by Moskowitz et al. [1] and Kim et al. [23] and lastly, abnormal performance results from Stoxx600 market. Section 5 discusses empirical results from the study, compares findings with the studies of Moskowitz et al. [1], He and Li [2], D’Souza et al. [4], Pitkäjärvi et al. [20] and Liu et al. [3] and indicates implications. Section 6 concludes and summarizes the overall finding of the study.

## 2 Literature review

### 2.1 Efficient market hypothesis and price anomalies

“… It turns out that Fama and Shiller agree more than they disagree. Both accept that markets are hard to beat by ordinary investors”∼ (Statman [24]; pp. 65–73).

The quotation above addresses several important questions that have been the subject of a wide debate among scholars since 1970. Fama puts forth the idea that financial markets cannot be beaten continuously. If stocks are always exchanged at their intrinsic market value, then it is virtually impossible to either buy undervalued stocks or sell overvalued stocks for earning abnormal returns. No one can do better than the performance of the overall market. If this is true, then the only way investors can generate extra profit is by taking greater risk, e.g., increasing market exposure. The key concepts behind Fama’s theory are that information is universally shared, people are rational and that stock prices follow a ‘random walk’. The strength of these assumptions depends on the form of “Efficient Market Hypothesis” (EMH) under consideration: weak, semi strong, or strong [14,25,26]. Jensen [15], along with a huge body of literature agree that “*the Efficient Market Hypothesis is virtually true … criticism is of limited value*”. Conversely, many empirical studies report findings on different types of securities' price anomalies [7,[27], [28], [29], [30], [31], [32], [33], [34], [35], [36], [37],^38^]. An anomaly is a deviated behavior of a financial market return that is inconsistent with EMH. If an anomaly is identified, then it could be possible to structure and implement a strategy to gain extra profit and therefore, outperform the market. Either testing the EMH or identifying an anomaly inextricably involve the application of asset pricing models to compare expected returns to real returns. As the ‘Joint Hypothesis Problem’ states, anomalous market returns may be a consequence of market inefficiency, inaccuracy in the pricing model or simply both [14]. What appears to be interesting is that market anomalies tend to vanish as soon as they are published in the economic literature: whether they disappear because of a sample selection bias or due to the behavior of market agents who exploit the anomaly trading accordingly is still doubtful [7,39]. An interesting survey that portrays a detailed list of price anomalies is presented by Schwert [7]. The author, through cross-sectional and times-series in asset returns, distinguishes anomalies in ‘predictable differences in returns across assets’ and ‘predictable differences in returns through time’.

### 2.2 From cross sectional momentum to time series momentum

Among the price anomalies studied in the survey of Schwert [7], two phenomena have gained particular attention: momentum effect or price continuation and winner loser reversal or price reversal or contrarian effect. Chronologically, the first anomaly to be documented was price reversal by De Bondt and Thaler [16] in a paper entitled ‘Does the Stock Market Overreact?’. Although this phenomenon was initially discovered in relation to behavioral finance, its practical relevance has been crucial: The psychological influence on financial markets leads to overreaction and, therefore, “*extreme movements in stock prices will be followed by subsequent price movement in the opposite direction*”. In the scope of tactical asset allocation this anomaly may be exploited by going short those assets that have shown extreme increases in prices and long those assets that have shown extreme decreases in prices to earn excess returns. This clearly violates the weak form of market efficiency. What appears to be interesting is the empirical approach of De Bondt and Thaler [16] adopted to identify the contrarian effect since many other empirical studies, thereafter, have followed the same methodology but using the opposite trading logic, i.e., momentum strategies (e.g., Daniel and Moskowitz [40] and D’Souza et al. [4]). To test the hypothesis that stock prices' extreme movements are followed by movements in the opposite direction. De Bondt and Thaler [16] computed cumulative abnormal returns over non-overlapping formation periods. After sorting each stock, they assigned the stocks in the upper decile to the winner portfolio, while the stocks in the lowest decile to the loser portfolio. If overreaction exists, then going long the loser portfolio and short the winner portfolio would entail profit. This was econometrically tested by computing the average cumulative abnormal return for the winner (ACAR_W_) and loser (ACAR_L_) portfolios. Consequently, the average profit would be equal to ACAR_L_–ACAR_W_.

This phenomenon was not only analyzed relative to individual stocks but also in consideration of national equity indices by Richards [41]. An additional insight provided by Richards is whether contrarian effect embodies time-varying risk. However, he did not find robust evidence that price reversal was caused by risk exposure and the anomaly was confirmed. This price anomaly was found to occur during a horizon of 3–5 years, that is the main difference and consistency with momentum effect. The latter seems to take place over a relatively shorter term. Conrad and Kaul [18] states that these two phenomena do not contradict although they seem to be ‘*diametrically opposed in theory and execution*’*.* Jegadeesh and Titman [17] use a similar model to that one implemented by De Bondt and Thaler [16] to test for momentum effect. The two main differences are the portfolio formation period and ratio behind the strategy. Jegadeesh and Titman [17] propose to go long stocks in the top decile (winners) and short stocks in the lowest decile (losers). By implementing this strategy, the study finds opposite conclusions as compared to De Bondt and Thaler [16]. After Jegadeesh and Titman seminal work, a huge body of literature confirmed the momentum effect. Among those studies few remarkable are as follows: Chan et al. [42], Carhart [30] and Van-Dijk and Huibers [43], etc.

Most of these studies are based on the model proposed by De Bondt and Thaler [16] and implemented by Jegadeesh and Titman [17] for momentum effect. However, Conrad and Kaul [18] suggest a different methodology where the momentum strategy’s profitability derives from cross-sectional dispersion in the assets' mean return. To examine the differences between cross sectional momentum strategy and time series momentum strategy, and their source of profit, three academic papers provide us with clear guidelines: ‘An Anatomy of Trading Strategies’ (Conrad and Kaul [18]), ‘Cross-Sectional and Time-Series Determinants of Momentum Returns’ [44] and ‘Time Series Momentum’ [1]. The first study allows us to outline the cross-sectional momentum strategy itself, the second study proves the low explanatory power of cross-sectional variance for momentum profit, and finally, the most recent study among the three aims at providing academic support to the econometric model used in the methodology section of this study, i.e., time series momentum. The empirical approach of Conrad and Kaul [18] differs considerably from other studies because they do not employ Jegadeesh and Titman’s methodology, i.e., selecting assets according to their realized return in the formation period, buying those that are in the top decile and selling those in the bottom decile (or vice-versa if it is a contrarian strategy, De Bondt and Thaler [16]. Conrad and Kaul [18] construct a portfolio by attributing to each asset the following weight as shown in equation (1):

(1) $$w_{i,t}\left(k\right)=\pm \frac{1}{N}\left[r_{i,t-1}\left(k\right)-r_{B,t-1}\left(k\right)\right]$$

where $w_{i,t}\left(k\right)$ is the weight of security i during the holding period t, $r_{i,t-1}\left(k\right)$ is the security’s return in the formation period t−1, $r_{B,t-1}\left(k\right)$ is the return on an equally weighted benchmark portfolio made on *N* securities in the period t−1 and *k* simply represents both the formation and the holding period. The right-hand-side of the equation may be positive or negative depending on the strategy, e.g., momentum or contrarian. Therefore, the more the security outperforms (underperforms) the benchmark portfolio over the formation period, the larger (lower) is the weight attributed to the security for the holding period. Hence, the investment, either it is long or short is the following as shown in equation (2):

(2) $$I_{t}\left(k\right)=\frac{1}{2}\sum_{i=1}^{N}\left|w_{i,t}\left(k\right)\right|$$

The sum of the weights will be equal to zero since the investor goes long in the assets with positive returns and short in the assets with negative returns. With those weights, therefore, it is possible to build an arbitrage portfolio (i.e., zero cost portfolio) as shown in equation (3):

(3) $$\pi_{t}\left(k\right)=\sum_{i=1}^{N}w_{i,t}\left(k\right)r_{i,t}\left(k\right)$$

where the profit of momentum strategy corresponds to the loss of price reversal strategy and vice-versa. Assuming stationarity of single asset returns, equation (4) tells that the total expected profits deriving from the strategy may be decomposed in two parts: asset returns' predictability through time series, assessed by P(k), and profits deriving by dispersion in mean returns of assets through cross-section, assessed by $\sigma^{2}\left(\mu \left(k\right)\right)$.

(4) $$\begin{array}{cc} E\left[\pi_{t}\left(k\right)\right] & =-Cov\left(r_{B,t}\left(k\right)-r_{B,t-1}\left(k\right)\right)\\ \; & +\frac{1}{N}\sum_{i=1}^{N}Cov\left(r_{i,t}\left(k\right)-r_{i,t-1}\left(k\right)\right)+\frac{1}{N}\sum_{i=1}^{N}{\left(\mu_{i,t-1}\left(k\right)\mu_{B,t-1}\left(k\right)\right)}^{2}\\ \; & =-C_{1}\left(k\right)+O_{1}\left(k\right)+\sigma^{2}\left(\mu \left(k\right)\right)\\ \; & =P\left(k\right)+\sigma^{2}\;\left(\mu \left(k\right)\right) \end{array}$$

In this equation, the term –C_1_(k) contains the negative autocovariance of the unconditional means between the asset *i* and of the benchmark and the term O_1_(k) reflects the average covariance of the selected portfolio. All put together, they refer to the predictability-profitability index P(k). While P(k) captures profit from predictability in time series returns, $\sigma^{2}\left(\mu \left(k\right)\right)$ captures the cross-sectional dispersion in the assets' means returns. To note that, if no correlation exists in individual or benchmark returns, the component P(k) becomes equal to zero. Still, due to cross-sectional dispersion in single assets' returns, the momentum strategy will provide positive returns.

After presenting the momentum profit decomposition, the Conrad and Kaul [18] conduct a bootstrap experiment to validate their finding: cross-sectional variance in mean returns by itself generates momentum profit. Through this simulation, they basically clean real data from time series relationships and, more in general, time series properties. The data ‘scrambling’ process deletes any autocorrelation in time series. The data set was ‘scrambled’ while keeping the other characteristics unaltered. The medium horizon strategies were simulated 500 times and the results appeared in line with their profit decomposition. Hence, they obtained a further confirmation that, between 1964 and 1989, cross-sectional variance alone justifies the profit coming from momentum effect. Additionally, from this experiment they note that the relationship between mean returns and holding period is in line with random walk theory. With 1-month formation period, the proportion of the average profit between 3-month, 6-month and 12-month momentum strategy is the same of what predicted by the random walk model: a geometric increase. Conversely, Jegadeesh and Titman [45] conduct a further study comparing their results to a portfolio with low dispersion in expected returns and found that cross sectional dispersion in assets' returns in not the only source of momentum profit, they say ‘*if there is any*’. Jegadeesh and Titman [45] conclude that Conrad and Kaul [18] rejected time series predictability because of a small sample bias. To achieve this conclusion, the authors implement an unbiased bootstrap test and find that the momentum profit is null. Cross-sectional variance is meaningless in generating profit. Also, the same authors argue that the evidence reported by Conrad and Kaul is in contrast with the assumption that momentum profit is generated by the time-series properties as the data was scrambled. The small sample bias takes place because the returns are drawn with replacement and therefore, the very same return observation may fall either in the formation or holding period. After, they formally show the biasness and propose a similar bootstrap test but this time, without replacement. The difference in the experiments is as equations (5) and (6) respectively:

(5) $$E\left(\pi_{rep,t}^{*}|r_{i,j},i=1,\ldots ,N,j=1,\ldots ,T_{i}\right)=\hat{\mu_{i}^{2}}-\bar{\mu^{2}}=\sigma_{\hat{\mu }}^{2}$$

(6) $$E\left(\pi_{no\;rep,t}^{*}|r_{i,j},i=1,\ldots ,N,j=1,\ldots ,T_{i}\right)=\frac{1}{N}\sum_{i=1}^{N}\mu_{i}^{2}-\bar{\mu^{2}}=\sigma_{\mu }^{2}$$

In these experiments, $r_{i,j}$ denote the time series of returns and $\pi_{rep,t}^{*}$ and $\pi_{no\;rep,t}^{*}$ denote respectively the momentum profit with and without replacement. The first equation was used by Conrad and Kaul [18] while the second by Jegadeesh and Titman [45]. By replicating 500 times the bootstrap without replacement Jegadeesh and Titman [45] finds that momentum profit, on average, is null and thus cross-sectional variation in expected returns is not the source of momentum profit. Their study supports behavioral explanations of momentum profit rather than the random walk theory as the previous two researchers point out. Thereafter, considerable number of studies are done on behavioral models were published to explain momentum effect and its impact on returns [43,46,47]. Relatively more recently the study by Moskowitz et al. [1] document a new form of momentum termed as time series momentum. For the future markets of equity indices, government bonds, currencies and commodities, their theory estimate the following simple equations (7) and (8):

(7) $$\frac{r_{t}^{s}}{\sigma_{t-1}^{s}}=\alpha +\beta_{h}\frac{r_{t-h}^{s}}{\sigma_{t-h-1}^{s}}+\varepsilon_{t}^{s}$$

and

(8) $$\frac{r_{t}^{s}}{\sigma_{t-1}^{s}}=\alpha +\beta_{h}sign\left(r_{t-h}^{s}\right)+\varepsilon_{t}^{s}$$

The study finds significant positive predictability from assets' own past 12-month returns for each of the future contracts over 25 years. After this persistent pattern, from the 13th month the trend reverses, i.e., winner loser reversal. Therefore, by looking back 1–12 months securities' own past returns, it is possible to buy winner assets and short loser assets, keep the position for the following 1–12 months and then close it, regardless of peer assets. While the literature cited thus far (i.e., Conrad and Kaul [18] and Jegadeesh and Titman [45]) mainly focuses on relative performances, e.g., cross-section, the finding of Moskowitz et al. [1] only look at the history of an individual asset’s return. This is consistent with sentiment theories: initial underreaction when there is positive time series momentum and then delayed overreaction when the series reverses over the long term. The first 12 months are positive predictors for future returns while after the 13th month, returns are negative predictors and often insignificant. In contrast to Conrad and Kaul [18], the outcome of Moskowitz et al. [1] contradicts the random walk model. If the random walk hypothesis implies that assets' own past returns are meaningless in predicting future returns, it seems that the price history matters since it may be employed to structure a trading strategy aiming at increasing alpha. They realize that a strategy so-structure not only generates an interesting reward-to-volatility ratio for each security but also that delivers significant positive alpha and low passive exposure to different types of factors. Interestingly, they find that during extreme market (S&P500) movements, the time series momentum strategy generates larger excess returns. Moskowitz et al. [1] also test the statistical significance of this finding and, indeed, the market squared is significantly positive. In their paper, they state that time series momentum strategy replicates the payoff of an option straddle. This is because the strategy is bullish when the market is hit by positive shocks and bearish when the market crashes. Finally, by running a factor regression adding proxies from the market of bonds and commodities, Moskowitz et al. [1] find a significant relationship between the excess return of time series momentum and cross-sectional momentum factor. The study analyzes this relationship by regressing time series momentum on cross-sectional momentum and decomposing cross-sectional momentum profit as described above. The study concluded that time series momentum actually subsumes cross-sectional momentum.

### 2.3 Evidence and criticism to time series momentum

The documentation of this new form of momentum effect (i.e., time series predictability based on momentum logic) triggered a new cycle of empirical studies aiming at producing evidence on time series momentum in some particular countries and/or within specific asset classes. Following Moskowitz et al. [1], numerous academic studies confirmed the existence of time series momentum both in developed and emerging economies. This section reports some of the most striking evidence that have contributed to highlight the occurrence of this price anomaly challenging the random walk hypothesis. He and Li [2] find significant and persistent time series momentum profit on the total return index of the S&P 500 for nearly 25 years, between 1988 and 2012. The study also proposes a behavioral model to explain time series momentum profit through market under and over-reaction. By using a stochastic delay integra-differential equation the study deployed both momentum and contrarian strategies simultaneously. The study shows that momentum strategies destabilize the market while contrarian trades restabilize it. Based on different trading rules, the model includes three types of agents: fundamental, momentum, and contrarian investors. He and Li [2] conjecture three market states: 1. Fundamental and contrarian traders are the main market players. 2. The activity of momentum traders is equally shared with fundamental and contrarian traders. 3. Momentum traders are the unique agents in the market. In market state 3, the TSM strategy is profitable during short periods and unprofitable during long periods, while it is never profitable in other states. The t-statistic of the mean return of the TSM strategy in 3rd state, from 1 to 60 months periods and different holding periods. The pattern is evident: at the beginning the t-values are significantly positive (i.e., own past returns are positive predictors) and after that, their values start to decrease until they become significantly negative (e.g., own past returns are negative predictors). This trend changes depending on the holding period under consideration.

D’Souza et al. [4] in their empirical study, documents almost 100-years (1927–2014) time series momentum on the outstanding number of individual stocks on the NYSE, American Stock Exchange and NASDAQ, and starting from 1975 they extended the research to 11 European countries. Like Moskowitz et al. [1], they show the t-values of the lagged returns' coefficients from 1 to 36 month. In terms of trend, this finding is perfectly coincident to Moskowitz et al. [1] and He and Li [2] - up to the 12th month regressors are positive predictors. However, differently from Moskowitz et al. [1] and D’Souza et al. [4] transform the variables of the regression equation in log scale. Zakamulin and Giner [48] conduct a study on time-series momentum in the US stock market and find that, in the short term, price continuation is in place. A long strategy based on time series momentum logic results to be profitable with a Sharpe ratio greater than 30% compared to a buy and hold strategy. They also explain that the probability that the TSMOM strategy outperforms the buy-and-hold strategy is less than 60% when the usual threshold is 80% arguing that this is the reason for many studies going against time series momentum. Hurst et al. [5] produce a further century of time series momentum evidence using monthly returns for 67 markets across different asset classes between 1880 and 2016: 29 commodities, 11 equity indices, 15 bond markets, and 12 currency pairs. Their time series momentum strategy generates positive Sharpe ratios for each security they take into account. In line with Moskowitz et al. [1], they find that in the greatest number of market crashes, time series momentum earns profit when the market collapses, like a straddle option payoff. They show the annual returns of time series momentum (gross of fee, net of cost) versus U.S. equity market returns, 1880–2016, as well as the fitted second order polynomial.

Georgopoulou and Wang [6] documents TSM of international mutual fund performance of emerging and developed markets, where emerging markets provide higher returns in TSM, compared with developed. However, TSM is of a shorter interval in emerging markets, while profitability of TSM strategy begins to be lost much faster than in developed markets. Considering the return continuation pattern in the currency component, returns from emerging markets are much higher compared with returns from developed markets. In some more recent study, Liu et al. [3] prove that between 2007 and 2019, time series momentum on 31 Chinese commodity futures contracts experienced several drawdowns. Based on tail-distributed upper and lower partial momentum, they build a decision function to forecast those drawdowns, they term this strategy ‘Managed time series momentum’. Consequently, they are able to enhance the reward-to-volatility and systematically reduce the level of drawdown. At the same time, Pitkäjärvi et al. [20] suggest an extension of Moskowitz et al. [1] time series momentum model to capture cross-assets signals between equities and bonds between 1980 and 2016. They find that past stock returns may be used to negatively predict future bond returns and past bond returns to positively predict future stock returns.

Liu et al. [3] also report the cumulative excess returns of a long passive strategy (LONG), time series momentum (TSMOM), and cross-asset time series momentum (XTSMOM) portfolios diversified across both bond and stock indices. Each strategy is structured with a lookback period equal to 12 months and a holding period of 1 month. The sample period goes from the beginning of 1980 to the end of 2016. The strategy implemented accordingly leads to a Sharpe ratio remarkably higher than a normal time series momentum strategy. The study finds that single asset time series momentum is significant and persistent for the first months and then the trend reverses as in previous findings. In terms of cross-assets signals, the authors find that crude oil options (USO) exhibit negative predictability for future stocks returns (e) in the short period and vice versa. It turns out that the cross-assets time series momentum strategy significantly outperforms all the strategies (B&H and TSM) with a mean excess return of 0.92% per month (11.04% per year), and with a Sharpe ratio of 0.29 per month. The cumulative returns starting from 1 dollar deriving from XTSMOM is indeed 3.47 against 1.73 from TSMOM. However, plotting the XTSMOM strategy excess returns against the TSMOM it appears that the latter produces a more convex line indicating that during more extreme market moves, it earns greater profit. Although the time series momentum is highly acknowledged by the earlier studies yet it is not immune to subsequent critics in addressing these two issues as mentioned below:

1. Volatility-scaling leads time series momentum to earn abnormal return,
2. The model implicitly assumes that premiums are the same across asset classes and countries by imposing a common intercept.

As far as the first critic is concerned, back in 2016, in the ‘Journal of Financial Markets’, there appeared an article entitled ‘Time Series Momentum and Volatility Scaling’. In this study, Kim et al. [23] find that significant alphas from time series momentum strategies are mainly driven by volatility-scaling, and unscaled TSM alphas are approximately equal to unscaled long passive strategies alphas. Kim et al. [23] gauge the potential effect of volatility scaling on time series momentum profits by simply comparing the strategy with risk parity approach and without against a buy and hold strategy with risk parity approach and without for 55 futures markets from 1984 to 2013. The study finds that an unscaled time series momentum strategy underperforms the unscaled buy-and-hold strategy. In contrast, in most cases a scaled time series momentum strategy does not significantly outperform a scaled buy-and-hold strategy. Indeed, Kim et al. [23] show that although between 1997 and 2011 time series with volatility scaling grew faster than buy and hold strategies with volatility scaling, at the end of the period (2013) the two strategies tend to converge. They additionally plot the alphas from the two different strategies with and without volatility scaling to compare relative abnormal performances. While it seems to be clear that individual alphas of time series momentum with volatility scaling are persistently larger than alphas of a buy and hold strategy with volatility scaling, they also report greater buy and hold strategy’ alphas without scaling for their ex-ante volatility. This is another evidence they find against time series momentum. Finally, the study concludes that profitability of time series momentum only depends on the risk parity approach that increases both Sharpe ratios and alphas of the portfolios.

The study by Huang et al. [19] examined the statistical reliability of the model implemented by Moskowitz et al. [1]. Through parametric and non-parametric bootstrap tests study invalidate the t-statistics obtained by Moskowitz et al. [1] in their pooled panel regression. However, Huang et al. [19] agree on the profitability of time series momentum strategies. Besides the pure technicalities to construct the bootstrap tests, the reason that led the authors to conduct this study is that Moskowitz et al. [1] implicitly assume that the mean returns of all securities are identical by imposing the same alpha. In contrast, the researchers show that the sample means of individual assets change dramatically across asset classes and, Jorion and Goetzmann [49] prove that the stock excess returns are different across countries. Consequently, they conclude that the model of Kelly et al. [50] is biased upwards. They use a parametric (*wild*) and a non-parametric (*pairs*) bootstrap to simulate the t-statistic and they also establish a confidence interval of 97.5%. Consequently, if the t-value from the real data is larger than 1.96 but lower than the simulated t-statistic, one can conclude that the pooled regression tends to over-reject the null hypothesis. They repeat this procedure 1000 times and attain the distribution of the *t*-statistic in order to test the null hypothesis (i.e., absence of time series momentum) in the sample period is 1985–2015. For instance, when predicting the return of next month with the past one year monthly returns scaling for the ex-ante volatility, the *t*-value of the regression beta range between 1.80 and 1.68. To sum up, the effect of volatility scaling as a risk parity approach as well as the assumption that securities' premia are the same across different asset classes and countries seem to converge in contradiction to the evidence provided by Moskowitz et al. [1]. However, if only an asset class from one single country is chosen for the pooled panel regression (as in this study), the limits to the time series momentum model discussed above should not apply. For example, Liu et al. [3] use respectively US equity indices and Chinese commodity futures for their time series momentum analysis and the outcome is consistent with Moskowitz et al. [1].

## 3 Data, methods and empirical testing

### 3.1 Data

The dataset under analysis consists of total return indices obtained from Bloomberg for national equity indices for most European countries.

**Fig. 1:**
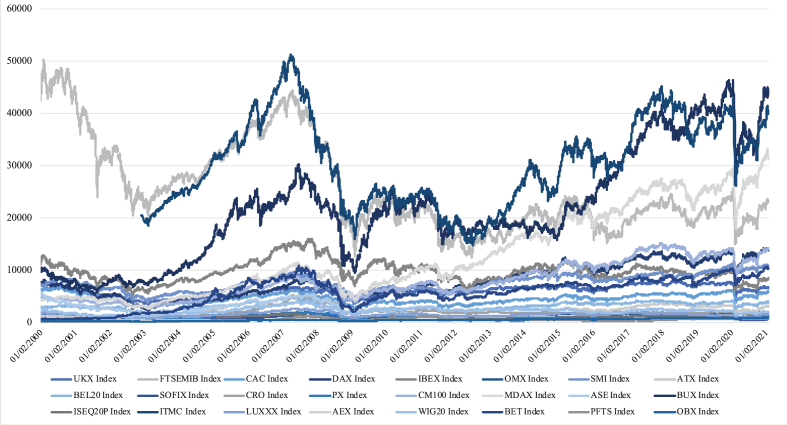
Plot series of the 24 European equity indices. Note: This figure shows the daily market prices of the 24 European equity indices used in the empirical study from Jan 2000 to the end of Jan 2021. However, only the time series until the end of 2020 has been used as input in the models.

The dataset also comprehends mid-cap stock indices for Germany, France and Italy since a huge number of liquid companies are traded under this category. Hence, we have 24 equity indices reported in Table 1. The sample period is from the 1st of January 2000 to the end of December 2020, for a total of 5040 daily observations for each national stock index as shown in Fig. 1. Table 1 shows some annualized descriptive statistics of the monthly returns' stock indices: country of origin, ticker, start date of the series, average return, standard deviation, Skewness, Kurtosis and Sharpe ratio calculated as the expected total return (E[r]) minus the European risk-free rate (EURIBOR), all divided through the standard deviation (σ[r]).

**Table 1:** Descriptive statistics.

Country	Ticker	Start date	E[r]	σ[r]	Skew	Kurt	Sharpe ratio
AUSTRIA	ATX	02/01/2000	0.0603	0.2072	−0.3062	6.7638	0.242761
BELGIUM	BAL20	02/01/2000	0.0456	0.1973	−0.1172	7.1869	0.180436
BULGARIA	SOFIX	24/10/2000	0.1037	0.2093	0.2072	32.7855	0.447683
CROATIA	CRO	14/06/2002	0.0378	0.1645	0.4892	20.8856	0.168997
CZECH REPUBLIC	PX	02/01/2000	0.0403	0.1762	−0.4027	9.8976	0.171964
FRANCE	CAC40	02/01/2000	0.0241	0.2074	−0.0004	5.9759	0.067985
FRANCE MID CAP	CM100	02/01/2000	0.0608	0.1375	−0.4573	4.9876	0.369455
GERMANY	DAX	02/01/2000	0.0637	0.1974	0.0027	4.8857	0.272036
GERMANY MID CAP	MDAX	02/01/2000	0.09724	0.1675	−0.1784	5.8974	0.520836
GREECE	ASE	02/01/2000	−0.0525	0.1894	−0.1089	4.8494	−0.32999
HUNGARY	BUX	02/01/2000	0.116	0.203	0.0063	3.8349	0.522167
IRELAND	ISEQ20P	03/07/2005	0.0431	0.1791	−0.3529	6.8392	0.184813
ITALY	FTSEMIB	02/01/2000	0.0007	0.2189	−0.3002	5.7839	−0.04485
ITALY MID CAP	ITMC	01/02/2003	0.0533	0.1507	−0.5242	8.8749	0.287326
LUXEMBURG	LUXXX	02/01/2000	0.0083	0.1786	−0.1627	3.8739	−0.00951
NEITHERLAND	AEX	02/01/2000	0.0141	0.1895	−0.0134	5.7383	0.021636
NORWAY	OBX	02/01/2000	0.0956	0.2186	−0.4082	4.8393	0.391583
POLAND	WIG20	02/01/2000	0.0195	0.2058	−0.1654	2.7333	0.046161
ROMANIA	BET	02/01/2000	0.1333	0.1892	−0.4628	13.8721	0.651691
SWEDEN	OMX	02/01/2000	0.0723	0.1822	0.0672	2.3876	0.341932
SPAIN	IBEX35	02/01/2000	0.0022	0.2006	−0.0431	5.8489	−0.03888
SWITZERLAND	SMI	02/01/2000	0.0247	0.133	−0.0972	5.1982	0.110526
UK	FTSE100	02/01/2000	0.01719	0.1561	−0.1282	4.8239	0.046060
UKRAINE	PFTS	02/01/2000	0.1719	0.2008	0.8727	16.0208	0.806275

*Note:* This table illustrates some features and descriptive statistics of the 24-time series from Jan 2000 to Dec 2020: country of origin, ticker, start date of the series, average return (E[r]) , standard deviation (σ[r]), Skewness, Kurtosis and Sharpe ratio of each European equity index of the data set. Average return, standard deviation and Sharpe ratio are annualized, and the Z-test suggests us that all these values are significant at 99%.

The monthly return series is used to run the pooled regressions outlined in the next paragraph as well as to implement the time series momentum strategy and the long passive strategy. In addition to this, one more monthly price series is exported from Bloomberg terminal as a proxy for the European market trend: the Stoxx600. The time horizon is identical to the previous dataset (i.e., 20 years). Finally, a dataset consisting of Fama & French five factors (MKT, SMB, HML, RMW, CMA) is downloaded from Kenneth French’s website^1^ as well as the cross-sectional momentum factor (WML) in European context.

### 3.2 Pooled autoregression

A pooled autoregression is used in order to produce empirical evidence on the predictability power of equity indices' own past returns. The analysis takes the heterogeneity explicitly into account by allowing for country-specific variables. It also contributes to more variability, degrees of freedom and efficiency as well as it is well suited to study the dynamics of change (motivated by Davidson and MacKinnon [21]. After having checked for stationarity through the Augmented Dicky Fuller Unit Root test and after all equity indices' returns and dates are stacked as a pooled dataset, the monthly return for index (s) in month (t) is regressed on its lagged h month, from h = 1 to h = 36 (3 years). Both the dependent and independent variables are scaled by their *ex-ante* volatility to have all returns in the same scale and make better comparisons among indices of different countries, this estimator is very similar to the Generalized Least Square [1] as shown in equations (9) and (10):

(9) $$\frac{r_{t}^{s}}{\sigma_{t-1}^{s}}=\alpha +\beta_{h}\frac{r_{t-h}^{s}}{\sigma_{t-h-1}^{s}}+\frac{\varepsilon_{t}^{s}}{\sigma_{t-1}^{s}}$$

where the *ex-ante* volatility is computed as follow:

(10) $$\sigma_{t}^{2}=252\sum_{i=0}^{\infty }\left(1-\delta \right)\delta^{i}{\left(r_{t-1-i}-{\bar{r}}_{t}\right)}^{2}$$

**Fig. 2:**
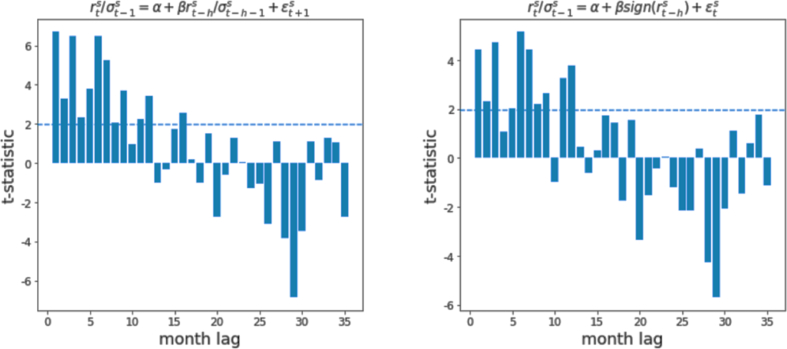
T-statistics and autoregressive model results. *Note:* The figures show the levels of T-statistics of the coefficients estimated using two different autoregressive model specifications as the formulas on top of the charts. In fact, in the horizontal axis the figures report the monthly lag returns from lag 1 to lag 35, i.e., 2 years and 11 months. These estimates have been computed on the 24-time series from Jen 2000 to Dec 2020 reflecting the different European equity indices.

The number 252 is to annualize the variance, $\left(1-\delta \right)\delta^{i}$ reflects the weight that sum to 1 and ${\bar{r}}_{t}$ is the weighted mean return estimated in a similar manner. The parameter δ is the one that coincides with the mass of $\left(1-\delta \right)\delta^{i}$ such that $\sum_{i=0}^{\infty }\left(1-\delta \right)\delta^{i}i=\delta /\left(1-\delta \right)$ = 60 days. Similar to Moskowitz et al. [1], D’Souza et al. [4] and Pitkäjärvi et al. [20], the study implements the same autoregressive model to assess the presence of time series momentum. In addition to equation (9), Moskowitz et al. [1] also propose another specification model using the sign of lagged returns as the independent variable (i.e., Equation (11)) for a robustness check in the time series momentum. The examination of this regression model highlights the tactical asset allocation strategy structured in the following paragraph. The second regression specification is attained with a small change in the right-hand-side of equation (9):

(11) $$\frac{r_{t}^{s}}{\sigma_{t-1}^{s}}=\alpha +\beta_{h}sign\left(r_{t-h}^{s}\right)+\varepsilon_{t}^{s}$$

where, by sign Moskowitz et al. [1] mean the sign function that equals +1 when r_t−h_ ≥ 0 and −1 when r_t−h_< 0. All the t-statistics of the coefficients for each lag are computed by clustering the standard errors by time (month) and plotted with lagged returns from 1-to-36 months. As the following chapter describes, the outputs across the two types of regression are quite consistent with the same trend (Fig. 2).

#### 3.2.1 Portfolio construction

The previous econometrics model is implemented to test the presence of time series momentum in the European equity indices. We now define the tactical asset allocation strategy that aims at quantifying the profitability deriving from this price anomaly. Like Goyal and Jegadeesh [51], Pitkäjärvi et al. [20] and Moskowitz et al. [1], this study proposes several strategies based on different lookback periods (k) and one month holding period (h). However, to conduct further analysis, it is used the conventional trading strategy in line with the cross-sectional momentum literature and consistent with the results of the previous regression model: a time series momentum strategy with 12 months lookback period and 1 month holding period (i.e., k = 12 and h = 1). For each equity index *s* and month *t*, the strategy goes long (short) if the return over the lookback period (i.e., the past 1-to-12 months) is positive (negative), then it holds the position for one month to close it immediately after. To size each position to hold for the following month, the strategy considers an ex-ante volatility equals to 20%. In other words, the size position will correspond to 20%/ $\sigma_{t}^{s}$. The 20% annual volatility is chosen as it roughly coincides with the average annual volatility of all equity indices (Table 2). This means that the more volatile indices have a lower weight in the scope of the portfolio than less volatile indices. This is done to obtain that the expected volatility in each position on the index is identical. Therefore, the strategy’s return for any equity index s at time t with volatility scaling similarly proposed by Moskowitz et al. [1] as shown in equation (12):

(12) $$r_{t,t+1}^{TSM,s}=sign\left(r_{t-k,t}^{s}\right)\frac{20\%}{\sigma_{t}^{s}}r_{t,t+1}^{s}$$

while without volatility scaling is represented by equation (13):

(13) $$r_{t,t+1}^{TSM,s}=sign\left(r_{t-k,t}^{s}\right)r_{t,t+1}^{s}$$

**Table 2:** TSM statistics.

Lookback period (h)	Return %		Volatility %		Sharpe ratio		Skew		Kurt		Max drawdown %		Colmar ratio %	
	TSM	B&H	TSM	B&H	TSM	B&H	TSM	B&H	TSM	B&H	TSM	B&H	TSM	B&H
**1**	8.17	2.42	12.17	16.55	0.70	0.23	2.32	−0.87	14.66	3.15	−16.91	−66.48	48.30	3.65
**2**	8.74	2.47	12.85	16.55	0.72	0.23	2.06	−0.88	11.91	3.15	−16.37	−66.48	53.42	3.72
**3**	10.36	2.59	12.36	16.54	0.86	0.24	1.68	−0.88	7.85	3.17	−13.35	−66.48	77.67	3.90
**4**	11.39	2.64	13.33	16.54	0.88	0.24	1.78	−0.89	10.50	3.18	−15.99	−66.48	71.22	3.97
**5**	12.13	2.68	13.37	16.53	0.92	0.25	1.48	−0.89	7.87	3.19	−21.82	−66.48	55.58	4.04
**6**	12.14	2.65	12.33	16.53	0.99	0.24	1.14	−0.89	3.99	3.18	−16.05	−66.48	75.62	3.99
**7**	12.62	2.62	12.94	16.53	0.99	0.24	1.27	−0.89	4.83	3.18	−16.89	−66.48	74.73	3.93
**8**	11.54	2.74	12.95	16.52	0.91	0.25	1.17	−0.89	6.61	3.21	−19.11	−66.48	60.42	4.13
**9**	10.75	2.77	12.28	16.52	0.90	0.25	0.60	−0.89	2.51	3.21	−21.30	−66.48	50.48	4.17
**10**	10.40	3.10	13.09	16.45	0.82	0.27	1.02	−0.91	5.30	3.32	−23.55	−66.48	44.18	4.67
**11**	11.01	3.08	12.87	16.45	0.88	0.27	0.86	−0.91	3.61	3.32	−23.24	−66.48	47.38	4.63
**12**	9.99	2.94	13.12	16.44	0.79	0.26	0.50	−0.90	2.37	3.32	−24.34	−66.48	41.04	4.42
**13**	7.74	3.24	12.79	16.39	0.65	0.28	0.36	−0.92	2.22	3.42	−32.76	−66.48	23.62	4.88
**20**	6.46	4.32	11.89	16.05	0.59	0.35	0.59	−0.95	2.42	3.82	−23.96	−66.48	26.95	6.49
**28**	4.63	3.45	12.40	15.96	0.43	0.29	0.61	−0.93	4.49	3.91	−41.83	−66.48	11.07	5.19

*Note:* This table reports statistics of the time series momentum (TSM) strategies related to 28 lookback periods (first column) in comparison to a buy and hold strategy (B&H): annualized average return, annualized volatility, annualized. Sharpe ratio, Skewness, Kurtosis, maximum drawdown and Colmar ratio. These statistics have been computed using the 24-time series from Jen 2000 to Dec 2020 reflecting the different European equity indices to implement the different trading strategies and all figures report a level of significance above 95%.

Hence, the final return of the strategy diversifying across all national equity indices (S_t_) with volatility scaling is represented by equation (14):

(14) $$r_{t,t+1}^{TSM}=\frac{1}{S_{t}}\sum_{s=1}^{S_{t}}sign\left(r_{t-k,t}^{s}\right)\frac{20\%}{\sigma_{t}^{s}}r_{t,t+1}^{s}$$

The conventional strategy (k = 12 and h = 1), as above-mentioned, is used to compare time series momentum performance with some benchmarks. A passive long strategy (B&H) with volatility scaling and some descriptive statistics of the time series (TSH) itself were chosen to obtain a relative valuation. The buy and hold strategy (B&H) with volatility scaling is structured as follow as shown in equation (15):

(15) $$r_{t,t+1}^{B\&H}=\frac{1}{S_{t}}\sum_{s=1}^{S_{t}}\frac{20\%}{\sigma_{t}^{s}}r_{t,t+1}^{s}$$

while without volatility scaling is represented by equation (16):

(16) $$r_{t,t+1}^{B\&H}=\frac{1}{S_{t}}\sum_{s=1}^{S_{t}}r_{t,t+1}^{s}$$

where the only difference with the TSM strategy is that the sign is not considered since the strategy prescribes to go long and hold the position for one month whatever is the sign of the return in the lookback period. Finally, the monthly return obtained from the different strategies allows us to make comparisons in terms of risk/return and other risk matrices: annualized average returns, annualized volatility, Sharpe ratio, skewness, kurtosis, cumulative returns, drawdown, and Colmar ratio. It provides a high-level interface for drawing attractive and informative statistical graphics.

### 3.3 Factor model

The study evaluates the abnormal performance from the time series momentum strategy with exposure to Fama and French [38] five factors and momentum factor as the sixth factor as shown in equation no. 17.

(17) $$r_{t}^{TSM\left(k,h\right)}=\alpha +\beta_{1}MKT_{t}+\beta_{2}{SMB}_{t}+\beta_{3}HML_{t}+\beta_{4}RMW_{t}+\beta_{5}CMA_{T}+\beta_{6}WML_{t}+\varepsilon_{t}$$

where *MKT* represents the returns on a Europe’s value-weighted market portfolio (i.e., Stoxx600) minus the U.S. one month T-bill rate. The next four variables are the standard Fama French European 5 factors and *WML* is additional cross-sectional European momentum factor. This equation primarily aims to quantify the value and the statistical significance of alpha, that is, the intercept that measures the return of the time series momentum strategy over and above market. Secondly, it allows us to identify any possible passive exposure to the European market factor $\left(\beta_{1}\right)$, and through the other coefficients (up to $\beta_{5}$) for the firm’s size effect, firm’s value effect, firm’s profitability, and firm’s investment attitude (e.g., conservative, or aggressive). Finally, the coefficient $\left(\beta_{6}\right)$ refers to the relationship between time series momentum returns and a portfolio made of winners minus losers with respect to past performance (i.e., cross-sectional momentum factor). Furthermore, by regressing the excess returns obtained from the time series momentum strategy and the buy-and-hold strategy on the Stoxx600 excess returns (as a proxy of the European stock market), we get a striking difference on the behavior of the two strategies relative to European market trend.

## 4 Results

### 4.1 Evidence of 1-year time series momentum

The results obtained from the two regression specifications defined in the previous chapter are robust and in line with literature reported in paragraphs 2.2 and 2.3: momentum effect in the first 12 months and signals of price reversal for the following 2 years. The graph in the left-hand-side of Fig. 2 exhibits a statistically strong trend within the first year, from the 1st month lag to the 12th month lag. All t-statistics are considerably above 1.96, except for the 10th month lag. The lags 1, 3, 6 and 7 are even above 5 suggesting very high predictability power for future returns. Similarly, the graph in the right-hand-side of Fig. 2 shows some little differences due to the different specification: while the previous trend is perfectly confirmed from the second model specification, all lags exhibit relatively lower t-statistics but still highly significant except the 4th month lag, that is now far above the 5% level of significance and lag 10, that is insignificantly negative. The pattern is identified by running these autoregressions coincides with the literature.

Moskowitz et al. [1] implement this model in relation to various asset classes: while the trend of government bond futures is blur; commodity futures, currencies and, especially, equity index futures provide us with very similar patterns. Also, in the study of Pitkäjärvi et al. [20] exhibits the same trend and t-statistics are lower too. However, the results shown in Fig. 2 are not to be taken for grant because R squared ranges between 9% and 24%.

### 4.2 Backtest of TSM strategies

The statistical evidence in favor of 12-months’ time series momentum reported in the previous paragraph is to have the statistical ground to implement the strategy described in paragraph 3.3 in order to exploit the upside of the price continuation phenomenon. Table 2 illustrates diverse statistics of the time series momentum strategy with volatility scaling (TSM) for each significant positive lag and a small sample of negative lags both significant and non, that is the lookback period (*k*), in comparison with a passive long investment strategy (B&H). These statistics provide a clear picture on the trade-off risk/return of time series momentum for European equity indices. Statistics comprehend annualized average return, annualized volatility, Sharpe ratio, skewness, kurtosis maximum drawdown and Calmar ratio. By construction, TSM strategies are based on the sign of the lookback period, as paragraph 3.2 explains, while for B&H strategies the lookback period is meaningless but aims at leveling the playing field for the comparison since the first returns of TSM, equal to the number of the lookback months, are skipped. What appears to be clear from the table below is that both the returns and the reward-to-volatility of the TSM strategy are remarkably larger than B&H strategy. By looking back 5, 6 and 7 months, one can obtain respectively 12.13%, 12.14% and 12.62% in annual return with Sharpe ratios nearly equal to 1. The relatively worst performances, instead, occur with lookback periods of 1 and 2 and 12 since returns and Sharpe ratios are respectively below 10% and 0.80. However, even the TSM’s worst performances are by far better than a B&H strategy. Cai & Schmidt 53 find that TSM has time periods in which investor capital is in cash and therefore investor portfolio during these times has zero returns. However, our results partially agree with them since the volatility is confirmed to be lower compared to a similar B&H strategy but expected return is systematically greater, *ceteris paribus*.

It is interesting to note that skewness and kurtosis of the return distributions suggest, for all TSM strategies, large possibilities to register extreme positive returns. Brooks and Kat [22] find that “*Sharpe ratios substantially overestimate the true risk-return performance of hedge funds*”. They state that “*Although hedge fund indices are highly attractive in mean-variance terms, this is much less the case when skewness and kurtosis are taken into account*”.

However, the backtest of TSM strategies provide the opposite results, making them more attractive for potential investors. While, if we consider the long passive strategy, the finding of Brooks and Kat [22] is consistent since skewness is negative across different lookback periods. Furthermore, the maximum drawdown for the TSM strategies oscillates between −23.55 and −13.35% while for B&H strategy is permanently equal to −66.48%. Considering the poor performance and the large maximum drawdown exhibited by the passive strategies, it is not unrealistic that Colmar ratios are below 5%. Conversely, TSM strategies have dramatically higher risk-adjusted returns as their Colmar ratios suggest. Finally, the last three strategies constructed with a lookback period corresponding to negative lags (13, 20 and 28) highlight how the more significant is the t-value the worse is the performance: Sharpe ratios are below 0.70. In these cases, a contrarian strategy would result as more profitable, that is, long loser assets and short winner assets would lead to a larger Sharpe ratio.

### 4.3 Performance of the conventional TSM strategy (l = 12, h = 1)

**Fig. 3:**
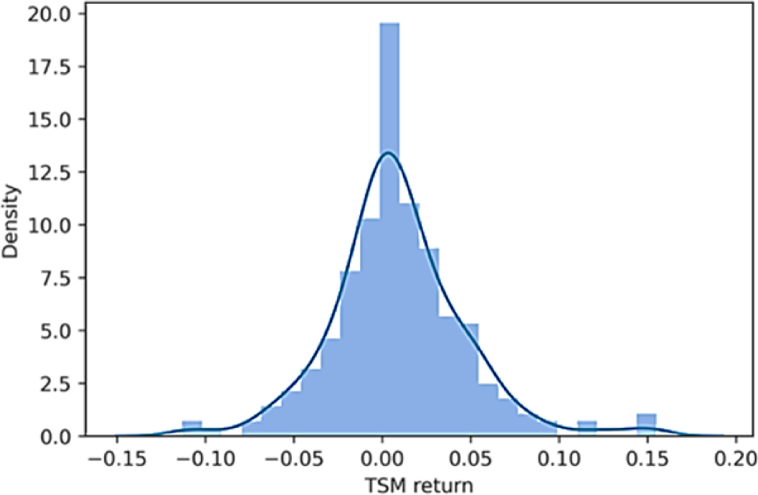
Monthly return distributions of TSM strategy. *Note:* This figure depicts the monthly return distributions from the time series momentum strategy (TSM) implemented using the 12 months as lookback period and 1 as month holding period. The equation that reflects the strategy is 3.6 and Table 3 shows quantitatively the returns for each month.

**Fig. 4:**
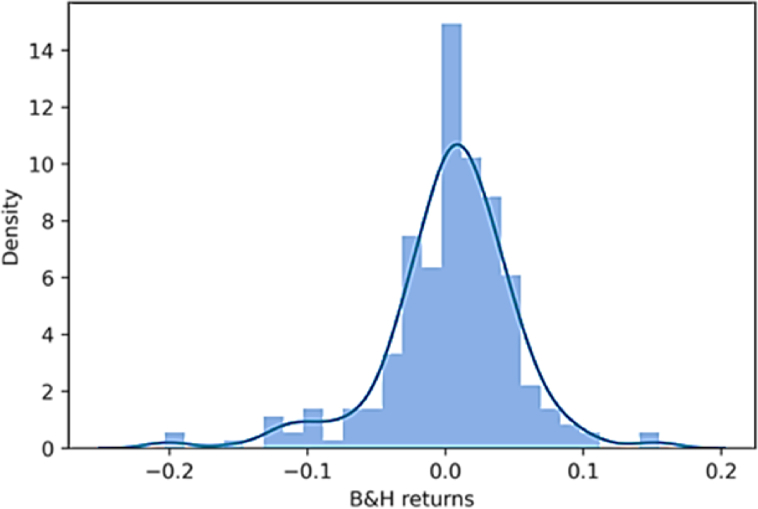
Monthly return distributions of B&H strategy. Note: This figure depicts the monthly return distributions from the buy and hold strategy (B&H). The look back period is meaningless while the holding period is 1 month again. The equation that reflects the strategy is 3.8 and Table 3 shows quantitatively the returns for each month.

The study conducts further analysis on the performance of the time series momentum strategy on the European equity market over the first 20 years of 2000, this section focuses on the most conventional strategy used in the cross-sectional momentum literature as well as the strategy used by Moskowitz et al. [1]: the properties of the 12-month time series momentum strategy with a 1-month holding period (e.g., k = 12 and h = 1). From Fig. 3 it is evident that the probability distribution of the monthly returns does not deviate much from a normal distribution. Returns far above the mean, dominate extreme negative scenarios, Skewness is indeed positive, i.e., 0.4957 (Table 2). Additionally, the negative excess Kurtosis indicates that the distribution has thinner tails (−0.6277). In contrast, the second histogram of Fig. 4 shows significant divergences of the empirical distribution of the ‘buy and hold’ strategy’ monthly returns from normality. In this case, there are possibilities to account for extreme negative returns since skewness is negative and excess kurtosis is positive.

To have a deep understanding of the monthly return distribution provided by the TSM strategy, it is interesting to observe Table 3 which portrays these returns for each year of the period considered.

**Table 3:**
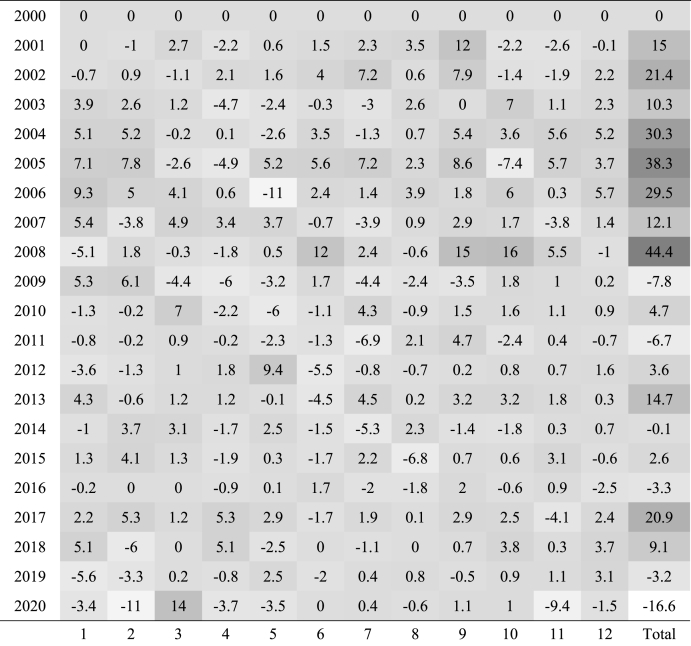
Monthly returns in percentage from TSM with K = 12 and H = 1.

*Note:* This table is created with ‘pyfolio’ Python library and reports the time series momentum strategy monthly returns for each year of the first 20 years of 2000, the Python library reports that all returns are significant at 99%. The horizontal axis represents the months while the vertical axis each year from 2000 to 2020. The difference between black and white determines whether the return is positive or negative and the grey tone the magnitude.

**Fig. 5:**
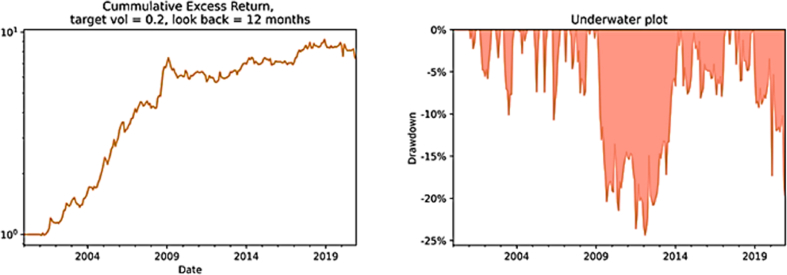
The cumulative returns and drawdown of TSM strategy. *Note:* The two figures portray the cumulative returns and drawdown of the time series momentum strategy (TSM) with a lookback period of 12 months and a holding period of 1 month, in the first 20 years of 2000s. The horizontal axis reports the different years while the vertical axis respectively the cumulative return investing 1 euro and the different levels of drawdowns.

The difference between black and white determines whether the return is positive or negative and the grey tone the magnitude. Although several squares are shown, especially in 2009, 2011 and 2020, the total column depicts 14 darker squares out of 20, where four of them are very dark (e.g., 2004, 2005, 2006, 2008). Such dark returns clearly represent positive outliers that skew the distribution leftwards (i.e., the positive skewness from Table 2). By conducting a visual inspection of Fig. 5, respectively the cumulative returns and the drawdowns over the 20 years considered, it is evident that the effects of those outliers are strong. The sharp uptrend of the time series momentum strategy interrupts at the end of 2008 when the negative consequences of the 2008 financial crises are absorbed by the strategy with some delay caused by the one-year lookback period. Since then, cumulative returns fluctuate until the end of 2020 producing the largest drawdown period of the strategy (2009–2014). The maximum drawdown occurs approximately in 2012 reaching −24.34% (also visible in Table 2). Afterwards, the crisis due to Covid-19 pandemic leads to one more important drawdown of the strategy as Fig. 5 shows. Overall, throughout the entire period the performance is good enough, especially if compared to a passive long strategy. In relative terms, the time series momentum strategy outperforms remarkably the ‘buy and hold’ strategy.

**Fig. 6:**
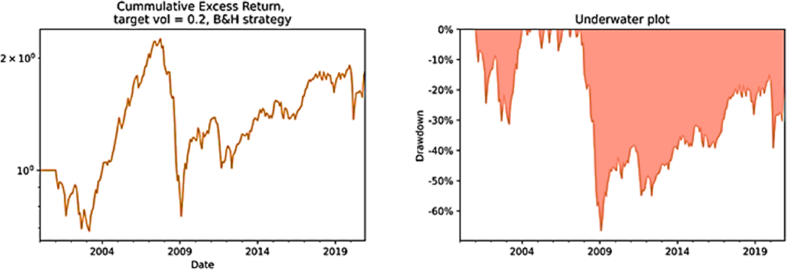
The cumulative returns and drawdown of B&H strategy. Note: The two figures portray the cumulative returns and drawdown of the buy and hold strategy (B&H) with a holding period of 1 month, in the first 20 years of 2000s. The horizontal axis reports the different years while the vertical axis respectively the cumulative return investing 1 euro and the different levels of drawdowns.

This was not only clearly visible in the statistics reported in Table 2, but also Fig. 6 confirm it. Cumulative returns are significantly more volatile and after the first big drawdown in 2008, it fluctuates vertiginously. Between 2009 and 2020 the passive long strategy continues to register huge drawdowns. The peak in cumulative return occurred in 2007 will never be reached again until the end of 2020. It seems to be clear that the monthly return distribution of B&H contains negative outliers that skew the distribution rightward. There must be some good chances to end up in extreme negative territory and this is reflected by the skewness equal to −0.90 (Table 2).Table

Since Kim et al. [23] find that unscaled time series momentum strategy underperforms an unscaled buy-and-hold strategy, and in most cases a scaled time series momentum strategy does not significantly outperform a scaled buy-and-hold strategy. The underlying study also computes Sharpe ratio, annualized return and annualized volatility in case of non-scaling for both TSM strategy and B&H strategy in order to compare them. According to Table 4, the TSM strategy back tested thus far (k = 12, h = 1) has been dramatically more attractive than a passive long strategy both with and without volatility scaling, although both strategies, when unscaled, underperform their scaled version at 5% level of significance.

**Table 4:** Volatility and strategies results.

	Return		Volatility		Sharpe ratio	
Strategy	scaled	unscaled	scaled	unscaled	scaled	unscaled
TSM (k = 12, h = 1)	9.99%	8.35%	13.12%	14.74%	0.793	0.616
B&H	3.90%	2.94%	15.91%	16.44%	0.322	0.261

*Note:* This table reports a comparison between volatility scaled and unscaled version of the two strategies, i.e., TSM with 12 months lookback period and 1 month holding period scale and unscaled versus B&H scaled and unscaled. The equation that represents the TSM strategy without scaling for the volatility is 3.5.

**Fig. 7:**
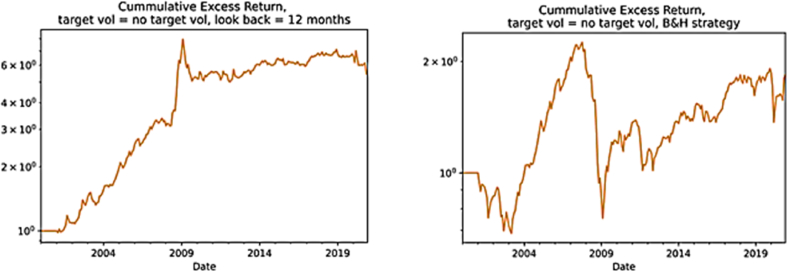
The cumulative returns from volatility-unscaled TSM and B&H strategies. *Note:* The first figure in the left-hand-side shows the cumulative returns from the TSM strategy volatility-unscaled while the second figure the cumulative returns from the B&H strategy volatility-unscaled. The horizontal axis reports the different years from 2000 to 2020 while the vertical axis the cumulative return if at the beginning of the period one would have invested 1 euro. The equation that represents the TSM strategy without scaling for the volatility is 3.5.

In terms of cumulative returns, the difference is substantial. We observe that TSM strategy volatility-unscaled produces considerably larger returns relative to B&H strategy volatility-unscaled (Fig. 7).

**Fig. 8:**
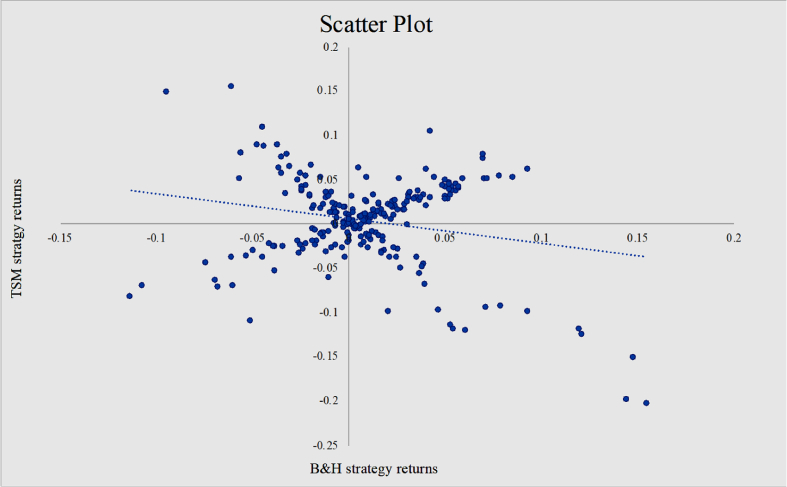
The scatter plot of TSM and B&H strategies. *Note:* This figure represents the scatter plot with the horizontal axis reporting the monthly returns from the buy and hold strategy and vertical axis the monthly returns of the time series momentum strategy with 12 months lookback period and 1 month holding period. It also portrays the regression line.

Furthermore, to gain additional insights on the performance of TSM strategy relative to the strategy of always being long, the excess returns from TSM strategy with a 12-month lookback period and 1 month holding period are regressed onto the returns from B&H strategy. As a result, alpha exhibits positive (0.98% per month) and significant (T-value = 3.97) and the slope is significantly equal to −0.1755. Therefore, such time series momentum strategy provides additional returns over and above a passive long position for portfolio diversified across European equity indices and it is negatively exposed to the passive long investment strategy (Fig. 8).

Thus far the analysis has been carried out in consideration of a TSM portfolio diversified across 24 European equity indices. The study continues with a comparison on the performance of TSM strategy, B&H strategy and historical time series (TS) for each of the countries composing the portfolio.

**Fig. 9:**
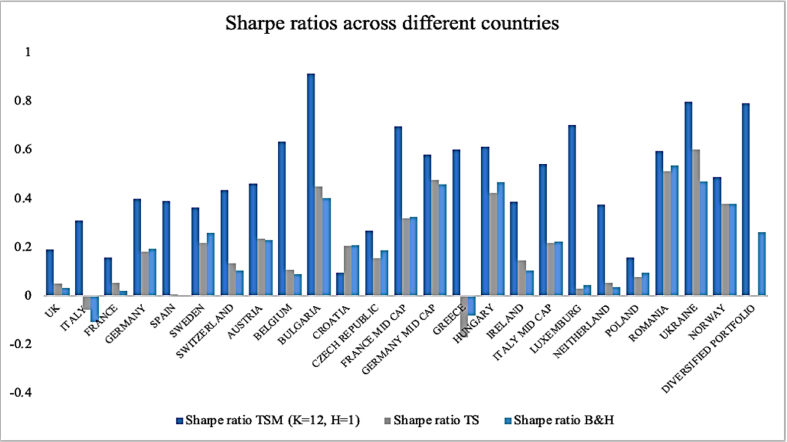
Sharpe ratios across different countries. *Note:* This figure illustrates the reward-to-volatility ratios from different strategies (i.e., Time Series Momentum with 12 months lookback period and 1 month holding period, Buy & Hold and historical time series itself) on each national equity index under analysis. Both numerator and denominator are statistically significant at 99% almost for all countries. We have the different European countries in the horizontal axis and the corresponding level of the Sharpe ratio in the vertical axis.

As Fig. 9 shows, for each country the TSM Sharpe ratio dramatically exceeds the other two, ranging between 0.09 (Croatia) and 0.91 (Bulgaria). By contrast, B&H and TS generate Sharpe ratios, on average, very close. Frequently, B&H is outperformed by the equity index itself in terms of reward-to-volatility ratio. These findings indicate that there are countries in Europe in which time series momentum is more pronounced and produce a better trade-off between risk and return. Moskowitz et al. [1] and Hurst et al. [5] also compare the Sharpe ratios obtained with time series momentum strategy among different asset classes and the equity instruments seem to lead to very similar results. However, they do not furtherly compare these results either to historical Sharpe ratios or buy and hold strategy Sharpe ratios.

### 4.4 Abnormal performance

In this section, the study evaluates the abnormal performance and control for passive exposure of the time series momentum’ monthly returns. Table 5 examines the risk-adjusted performance of the diversified TSM strategy with k = 12 and h = 1 strategy to the standard Fama & French five factors' exposures in comparison to the B&H. TSM delivers a positive and significant alpha of about 0.71% per month. At the same time, it does not exhibit significant betas on the market (MKT), SMB, HML, RMW and CMA but it has a significant relationship with WML, albeit the coefficient is close to zero.

**Table 5:** Results of regression.

		Alpha	MKT	SMB	HML	RMW	CMA	WML	R squared
TSM	Coefficient	0.71%	−0.0004	−0.0012	0.0009	−0.0008	0.0007	0.005	23.40%
	T-value	2.982	−0.747	−0.977	0.614	−0.447	0.369	7.097	
B&H	Coefficient	0	0.0068	0.0001	0.0046	0.0017	−0.0046	−0.0009	79.50%
	T-value	−0.023	19.747	0.191	4.919	1.478	−3.577	−1.844	

*Note:* This table reports the output of the regression model specified in paragraph 3.3 where the time series momentum strategy returns and the buy and hold strategy returns are the dependent variables respectively. In particular, this table shows the level of significance and the value of the coefficients for the intercept, the Fama-French and cross-sectional European factors (MKT, SMB, HML, RMW, CMA and WML) as well as the R squared value.

**Fig. 10:**
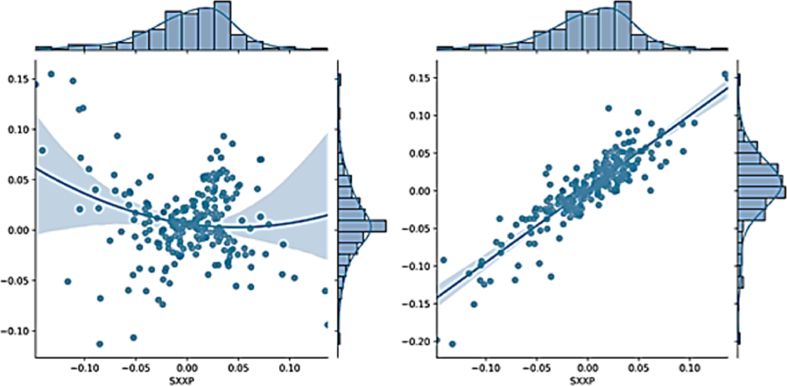
Scatter plots of TSM excess returns. *Note:* These figures depict the two scatter plots of time series momentum excess returns (vertical axis, first chart) and Stoxx600 excess returns (horizontal axis, first chart) with second order approximation curve and scatter plot of buy and hold excess returns (vertical axis, second chart) and Stoxx600 excess returns (horizontal axis, second chart) with the regression line.

Conversely, as Table 5 shows, the passive long strategy does not deliver abnormal returns. This strategy, instead, loads significantly positive on the market and HML while negative on CMA. In other words, a TSM strategy earns positive abnormal return while being neither exposed to the market nor to other factors, with exception of the cross-sectional momentum factor (WML). In more general terms, Fig. 10 highlights the different relationships between TSM excess returns and B&H excess returns against the Stoxx600 excess returns as proxy for the overall market. What seems to be clear from Fig. 10 is that the relationship between TSM and the Stoxx600 looks like an option straddle payoff on the market. Indeed, a convex curve (i.e., fitted second order polynomial) approximates more significantly the relationship between TSM and Stoxx600: the independent variable squared (excess market return squared) comes with a coefficient of 0.645, a t-value of 16.08 and R^2^ is 52.10%.

If the independent variable is not squared, the model does not replicate much of the variability since R^2^ amounts to 4.3%, alpha is significantly equal to 0.94% but the t-value is lower compared to the previous result. This means that when the market oscillates vertiginously, either positively or negatively, the strategy is more likely to generate larger profit. In contrast, the passive long strategy seems to follow the market trend. The first order line that interpoles all points is positively sloped (beta = 0.91 and t-value = 26.36) and the model replicates a lot of the variability (R^2^ = 74.6%).

This is a relevant result both from a practical and a theoretical point of view because it shows that market risk or crash risk does not completely offset the returns generated from the implementation of a time series momentum strategy. When the market experiences bad times, TSM shorts risky assets, and when it crashes, the strategy is likely to earn profit. Clearly, when the market rebounds, the strategy goes long risky assets and performs well. However, Fig. 10 also shows that the TSM strategy may underperform the market sometimes but the fitted second order polynomial suggests that this is less likely.

## 5 Empirical discussion and implications

Although Schwert [7] argues that many ‘price anomalies’ or ‘market inefficiencies’ seem to disappear after they are documented in the finance literature (e.g., the size effect and the weekend effect), the empirical evidence in favor of ‘time series momentum’ has been strong since its first documentation [1]. Either time series momentum should have vanished because of a sample selection bias or due to actions of practitioners who learn about the anomaly and trade accordingly, this particular form of market efficiency remained persistent across different asset classes, countries and intervals of time. Hurst et al. [5] backtest a time series momentum strategy with 12-month lookback period and 1-month holding period (k = 12, h = 1) for different asset classes (equity, bonds, commodities and currencies) between 1880 and 2016, and find that such strategy is significantly profitable and that it follows a straddle option payoff on the market; D’Souza et al. [4] prove time series momentum through the pooled panel regression (paragraph 3.1) from 1927 to 2014 for US individual stocks and some European stocks. For the interval 1980–2016, Pitkäjärvi et al. [20] conclude that time series momentum may be used for cross assets predictions between equities and bonds for 20 developed countries. He and Li [2] demonstrate the existence of time series momentum with statistically strong t-statistics for the S&P 500 in the period 1988–2012. More recently, Liu et al. [3] exhibit a new type of strategy, the term ‘managed time series momentum’, which aims at reducing the drawdown of the time series momentum itself. These authors used Chinese future commodities for a relatively smaller sample period (2007–2019). Finally, this study demonstrates further 20 years of time series momentum evidence starting from January 2000 to December 2020 for the equity indices of European countries including the United Kingdom and Switzerland. The results of the pooled autoregression in this research are in line with the previous literature. The trend of the coefficients' t-values for each month lag are very similar to Moskowitz et al. [1], D’Souza et al. [4] and Pitkäjärvi et al. [20].

What follows is that the influential survey on price anomalies of Schwert [7] could be upgraded of time series momentum under the category ‘predictable differences in returns through time’. The present empirical study furtherly exploits the finding of time series momentum statistically obtained through the pooled autoregression in order to structure a tactical asset allocation strategy (similar to Moskowitz et al. [1] for enhancing the alpha of portfolios diversified across various European equity indices. In terms of reward-to-volatility, the backtest of such strategy shows results similar to Moskowitz et al. [1] and Hurst et al. [5]. The Sharpe ratios of the diversified portfolios constructed with strategies using different lookback periods ranges between 0.70 to nearly 1, while at single country level the average Sharpe ratio is 0.45 with two of them being slightly negative. Furthermore, in contrast to Kim et al. [23], the performances of the strategies, both time series momentum and buy and hold with and without volatility scaling show the same results: TSM strategy outperforms B&H strategy in both cases. However, cumulative returns obtained in this study are lower compared to other papers. With European equity indices in the first 20 years of 2000s, cumulative return of time series momentum strategy (k = 12, h = 1) with volatility scaling is considerably below that of Moskowitz et al. [1], D’Souza et al. [4], Pitkäjärvi et al. [20] and Liu et al. [3]. The reason behind the worse single country Sharpe ratios and lower cumulative return is perhaps linked to the turbulent sample period chosen for the analysis. Between 2000 and 2020 the global economy was hit by the dot-com bubble (2000–02), the financial crisis (2007–08), the European sovereign debt crisis (2012–13), particularly strong in Europe, and the Covid-19 pandemic (2020). The choice to consider the first 20 years of 2000s in this study, was intentional with the goal to have more recent evidence on the performance of the European time series momentum, especially when the market is volatile. Therefore, this study produces empirical evidence related to a relatively new form of momentum effect, i.e., time series momentum, throughout the 2000s in Europe. The objective beyond it is to contribute to the wide debate on the efficiency market hypothesis under both a theoretical perspective and a practical point of view. Two points deserve to be cited regarding the theoretical relevance of the present research question. First, both the econometric evidence and the profitability of the time series momentum strategy seem to hardly challenge the efficient market hypothesis (EMH) and second, ‘Joint Hypothesis Problem’ suggests us that we are not certainly in front of a market anomaly because the goodness of the EMH is not testable *per se*. In terms of practical relevance, the underlying findings provide some clear guidelines for traders and investors who are interested in increasing abnormal performances without loading on other risk factors.

## 6 Conclusion

The study finds that nearly all 12-month lag returns of the 24 most traded European equity indices are positive predictors of future returns. Therefore, it is structured and backtest a time series momentum strategy with the goal to quantify the performance of a time series momentum strategy and with look back periods equal to the number of month lag returns that exhibit significant coefficient, i.e., the first 12-month lags, and one month of holding period. It turns out that each of the 12 strategies is profitable. Also, a time series momentum strategy on a non-diversified portfolio, i.e., implemented to individual European countries results positive. Furthermore, study compares these results to a long passive strategy, e.g., a buy and hold, and find that time series momentum strategy is far more attractive in mean-variance terms.

It appears to be more interesting, that the strategy based on this finding produces significant abnormal returns, even when compared to a buy and hold strategy, and it is not exposed to other risk factors like the market (Stoxx600) or Fama & French five factors, except for the cross-sectional momentum factor. Indeed, a second order polynomial curve approximates more significantly the relation between market excess returns and time series momentum strategy excess returns. This curve, in fact, looks like a straddle payoff, which means time series momentum strategies earn more profit during more pronounced market movements. From a theoretical perspective, these findings hardly challenge the efficient market hypothesis, while practically speaking they provide clear guidelines to investors who desire to increase portfolios' alpha. However, a few limitations of the study should be mentioned both in theory and practice. The risk parity approach (or volatility scaling approach), as described by Kim et al. [23] may lead to misleading results in terms of risk-return trade-off but to be in line with previous literature and to produce consistent results, we applied it to both the TSM and the B&H strategies to obtain comparable results. The study does not consider transaction costs which may be wide when implementing active tactical asset allocation strategies, such as TSM. Finally, in this paper we do not provide readers with clue and suggestions on how to enter the market with TSM strategies but we only report evidence to strengthen the literature regarding this type of price anomaly.

Further studies could investigate the behavioral causes that entail this phenomenon in the scope of market inefficiencies in the European equity market and especially in the 2000s, in order to produces significance about the *time-to-market* and a more rigorous method to approach these markets. The significance of further studies could be found if the forecasted data for the portfolio were used after the 12-month lag, using machine learning techniques (like forecasted Sharpe ratios in studies of Vukovic et al. [52].

## Author contribution statement

MoinakMaiti; Darko Vukovic; SalvatoreIngenito: Conceived and designed the experiments; Performed the experiments; Analyzed and interpreted the data; Contributed reagents, materials, analysis tools or data; Wrote the paper.

## Funding statement

This paper has been supported by the RUDN University Strategic Academic Leadership Program.

## Data availability statement

Data will be made available on request.

## Declaration of interest’s statement

The authors declare no conflict of interest.

## Footnotes

1 https://mba.tuck.dartmouth.edu/pages/faculty/ken.french/data_library.html.
